# Effects of two-year adapted physical exercise program and nutritional counselling on cardio-sarcopenia syndrome in older adults with low muscle function

**DOI:** 10.1186/s11556-025-00377-8

**Published:** 2025-07-04

**Authors:** Giovanna Pelà, Sara Tagliaferri, Elisa Adorni, Marina Aiello, Marco Salvi, Irene Zucchini, Riccardo Calvani, Emanuele Marzetti, Fulvio Lauretani, Giampaolo Niccoli, Marcello Maggio

**Affiliations:** 1 University of Parma, Parma, Italy; 2 University-Hospital, Parma, Italy; 3 Catholic University of the Sacred Heart, Rome, Italy; 4 Fondazione Policlinico Universitario Agostino Gemelli IRCCS, Rome, Italy

**Keywords:** Frailty, Sarcopenia, Physical exercise, Cardio-sarcopenia syndrome, Cardiovascular aging, Cardiac remodeling

## Abstract

**Background:**

Frail, sarcopenic older individuals have high risk of cardiovascular events and require multidomain interventions. The cross-talk between cardiac and skeletal muscle mass is crucial to maintain physical independence in this specific population.

The aim of the study was to evaluate, in a selected sample of frail, sarcopenic older adults, the influence of a two-year multimodal intervention, composed by exercise program and nutritional counseling, compared to a lifestyle education program, on echocardiographic parameters and the relationship between left ventricular mass and skeletal muscle.

**Methods:**

One-hundred subjects, among those enrolled in the SPRINTT trial at Frailty Clinic of the University-Hospital of Parma, underwent cardiac examination as part of the ancillary protocol CARDIOSPRINTT. Eighty-two participants completed the protocol and attended the final visit after approximately 25 months from enrolment.

**Results:**

We did not find significant changes in the intervention group compared with the control one. However, we captured the longitudinal effects of cardiovascular aging, including the reduction of left ventricular volumes and an impairment of systolic and diastolic function of both ventricles. We found a significant relationship between left ventricular mass and skeletal muscle mass, suggesting the existence of cardiac-skeletal muscle axis. This relationship was not independent of age, body mass index or systolic blood pressure.

**Conclusions:**

Our findings suggest that among frail, sarcopenic patients with different degree of mobility impairment, a multimodal intervention is necessary to improve cardiac health and counteract the cardiovascular aging. Modulating the cardiac-skeletal muscle axis may represent a novel and promising target for preventing cardio-sarcopenia.

**Supplementary Information:**

The online version contains supplementary material available at 10.1186/s11556-025-00377-8.

## Background

Cardio-sarcopenia is a clinical condition referring to the existing relationship between cardiac mass and skeletal muscle mass (SMM) during aging. Keng and coworkers were the first hypothesizing a link between SMM and cardiac muscle in a sample of sarcopenic older adults with lower left ventricular mass (LVM) [1].

In this context, we recently showed, in a selected sample of older people with low SMM and physical frailty, a positive correlation between SMM and LVM confirming the important interaction between sarcopenia with cardiac mass across aging [2].

The interaction between cardiac and SMM plays a crucial role in preserving physical independence in frail, sarcopenic older adults. The parallel age-related changes in cardiac and skeletal muscle could be specific targets for better understanding of cardiovascular (CV) aging and offer new perspectives for interventional strategies aimed at preventing or treating CV disease in older people [1–3].

Physical exercise is a potential deterrent of cardio-sarcopenia syndrome, given its ability to beneficially modulate both cardiac and skeletal muscle function through different mechanisms [4–7]. However, evidence on the cardiac effects of physical exercise, specifically in frail and sarcopenic older adults, remains lacking. No data are also available about the relationship between cardiac muscle and skeletal muscle, as well as how this link evolves over time.

The opportunity to clarify this topic has been offered by the CARDIOSPRINTT study, an ancillary study of the Sarcopenia and Physical fRailty IN older people: multi-componenT Treatment strategies (SPRINTT) project [8–10]. It was a randomized control trial conducted in frail, sarcopenic older subjects, aged 70 years or older, which aimed at investigating the effects of a multicomponent intervention (MCI; intervention group) based on physical exercise and nutritional counselling, administered for up to 36 months, on mobility disability prevention, compared to a healthy ageing lifestyle educational program (HALE; control group) [8–10]. The SPRINTT trial demonstrated that MCI was associated with a reduction in the incidence of mobility disability without sex-differences and with a significant increase of short physical performance battery (SPPB) score [10].

However, in this trial, the effect of this MCI on the CV system of frail, sarcopenic and older subjects has never been analyzed.

Thus, the main objective of the longitudinal CARDIOSPRINTT analysis was to investigate the effects of two-year multimodal intervention on echocardiographic parameters of a group of older adults enrolled in the SPRINTT trial.

As secondary aim, we addressed the impact of this intervention on the relationship between cardiac and SMM, embodied in the cardio-sarcopenia syndrome.

## Methods

### Study design

CARDIOSPRINTT [2] study was an ancillary protocol of the SPRINTT project, a randomized controlled trial [8, 9]. In addition to the main protocol, it adds a complete cardiac assessment including clinical evaluation with 12-lead resting electrocardiogram (ECG), conventional and Doppler tissue echocardiographic (DTE) examination, at the time of enrollment and after approximately 24 months. A detailed description of the echocardiographic examination is provided in the Supplementary Material.

Blood pressure (BP) and heart rate (HR) (OMRON 705 IT) were averaged from three consecutive measurements for each. 

Detailed descriptions of MCI and HALE are provided in previously published articles and in the Supplementary Material [7, 8, 11].

At baseline, multimorbidity was evaluated through the history of medical events (number of medical events), as well as the adverse events occurred during the study period were considered at follow-up.

Level of physical activity before the enrollment was assessed by a questionnaire providing detailed information on its type, intensity, and duration, in three periods of their life: from 20 to 40 years of age, from 40 to 60 years of age, and in the last year [11].

The CARDIOSPRINTT study was approved by the AVEN Local Ethics Committee (Identifier: 82/2016/SPER/AOUPR*).* Written informed consent was obtained from all participants.

The post hoc longitudinal analysis of the CARDIOSPRINTT study, which deriving from SPRINTT project, retains its randomized controlled trial design and is well-described in the paper.

### Study population

One-hundred community-dwelling, physically frail, sarcopenic subjects, aged 70 years and older, without significant heart disease, were enrolled from February 2017 to November 2017 in the Frailty Clinic of the University of Parma, one of the Italian site of the SPRINTT study [8–10]. Inclusion and exclusion criteria, as well as SMM and frailty assessment, are detailed in previously published articles and reported in the Supplementary Material [8, 9].

### Statistical analysis

All data are presented as mean ± standard deviation (SD) or median [interquartile range range [IQR]], as appropriate. We compared distributions of variables in two groups through Student t-test (independent data) or Mann-Whitney test U for continuous variables. Paired Student t-test or Wilcoxon test were performed to evaluate differences between baseline and follow up. Differences between the two groups or between time points of qualitative variables were evaluated through chi-squared and McNemar tests, respectively. The evaluation of changes of left and right structural and functional cardiological end-points from baseline to follow-up (time factor as intra-subject factor) and in MCI and HALE group (group factor as inter-subject factor) was performed through a mixed effect analysis of variance (ANOVA). This was controlled for differences in age, body mass index (BMI), systolic BP (SBP) and sex (analysis of covariance, ANCOVA).

Baseline to follow-up difference of physical end-points (gait speed, metabolic equivalent of tasks -METs-, steps) between the two groups were investigated through Mann-Whitney U test, while Wilcoxon signed-rank test was performed to explore baseline to follow up difference of physical end-points in MCI and HALE.

*P*-values ≤ 0.05 were considered as significant. Statistical analyses were performed through the software SPSS Statistics 29 (IBM, Inc. Chicago, IL, USA).

## Results

### Population characteristics at baseline

At baseline, one-hundred subjects were selected and underwent the cardiological evaluation. Of them, 18 voluntarily abandoned the study or died and eighty-two completed the protocol and attended the final visit at follow-up, after an average of 25 months (25.3 + 2.9 months): 46 (56%) were randomized to MCI, 36 (44%) to HALE, and were included in this analysis.

Table 1 shows the main characteristics of the studied population: 57 women, 25 men, and a mean age of 78.8 ± 5.3 years. The two groups at baseline were homogeneous for age, gender and height. The mean BMI was 27.4 ± 4.8 kg/m^2^, with a greater percentage of subjects randomized in MCI having BMI values greater than 30 kg/m^2^. SBP values ​​were significantly higher in HALE compared with MCI, without significant differences in diastolic BP (DBP) in the two groups.

**Table 1:** Baseline clinical characteristics of population

Characteristics	Total *n* = 82	MCI *n* = 46 (56%)	HALE *n* = 36 (44%)	*p* value	*P* value (age-adjusted model)
Age (y)	78.8 ± 5.3	78.4 ± 5.0	79.3 ± 5.6	ns	ns
Female No. %	57 (70)	35 (76)	22 (61)	ns	ns
Male No. %	25 (30)	11 (24)	14 (39)	ns	ns
BMI (Kg/m^2^)	27.4 ± 4.8	28.8 ± 5.2	25.6 ± 3.6	**0.003**	**0.004**
Weight (kg)	68.6 ± 14.0	71.6 ± 15.6	64.9 ± 10.6	**0.03**	**0.037**
Height (cm)	158.3 ± 8.1	158.2 ± 8.5	158.6 ± 7.6	ns	ns
HR (bpm)	65.7 ± 10.4	66.5 ± 11.1	64.6 ± 9.6	ns	ns
SBP (mmHg)	138 ± 19	134 ± 18	144 ± 18	**0.027**	**0.034**
DBP (mmHg)	79 ± 9	80 ± 10	79 ± 8	ns	ns
Smoking No. (%)	5 (6.1)	4 (8.7)	1 (2.8)	ns	ns
Previous smoker No. (%)	23 (28)	10 (21.7)	13 (36.1)	ns	ns
Physical Frailty and Sarcopenia					
Appendicular lean mass (Kg)	16.7 ± 3.7	16.6 ± 4.0	16.8 ± 3.3	ns	ns
Appendicular lean mass/BMI	0.61 ± 0.13	0.58 ± 0.12	0.66 ± 0.11	**0.05**	ns
SPPB score	7 [7–7.5]	7 [7–7.3]	7 [7–8]	ns	ns
Clinical Characteristics					
History of Medical Events (n°)	49 [47–51]	50 [47–51]	49 [47–51]	ns	ns
Hypertension, No. (%)	55 (67)	31 (67)	24 (67)	ns	ns
Diabetes mellitus, No. (%)	6 (7)	3 (7)	3 (8)	ns	ns
CAD, No. (%)	8 (10)	5 (11)	3 (8)	ns	ns
COPD, No. (%)	7 (9)	3 (7)	4 (11)	ns	ns
Therapy					
Beta-blockers, %	40.2	50	27.8	ns	ns
ACE inhibitors, %	30.5	28.3	33.3	ns	ns
ARB, %	23.2	28.3	16.7	ns	ns
Diuretics, %	24.0	28.3	19.4	ns	ns
Other medication^a^, %	22.0	28.3	13.9	ns	ns

Data are expressed as mean ± standard deviation, as median [interquartile range], or as number of subjects with corresponding percentage

*p* values refer to comparisons between MCI and HALE groups. Values marked as “ns” indicate non-significant differences (*p* > 0.05). Statistically significant differences (*p* < 0.05) are highlighted. Age-adjusted *p* values were calculated using multivariable models controlling for age

*ACE* angiotensin-converting enzyme; *ARB* angiotensin receptor blocker; *BMI* body mass index; bpm: beats per minute; *CAD* coronary artery disease; *COPD* chronic obstructive pulmonary disease; *DBP* diastolic blood pressure; *HR* heart rate; *IQR* interquartile range; *SBP* systolic blood pressure

^a^Other medications related to cardiovascular system

ANOVA and logistic regression analysis were performed to assess the age-related differences in clinical data at baseline, in both all population and the two groups. The age-adjusted model did not show any significant difference between MCI and HALE before intervention, except for BMI (*p* = 0.004), weight (*p* = 0.037) and SBP (*p* = 0.034) (Table 1).

There were no significant differences between the two groups in relation to comorbidities and pharmacological therapy. About the comorbidities, 67% had a history of hypertension, 7% had diabetes, 10% had coronary artery disease (CAD) and 9% chronic obstructive pulmonary disease (COPD). At the time of enrollment, all subjects were stable from a hemodynamic point of view, and none had heart failure (Table 1). The median number of medical events in all subjects was 49 [47–51], without any difference between the two groups (Table 1).

About 40% of participants were chronically on beta-blockers, 30.5% on ACE inhibitors, 23.2% on Angiotensin II receptor blockers (ARB), 24% on diuretics and 22% on other medications related to cardiovascular system (Table 1 and Supplementary Information for further details).

Regarding physical frailty and sarcopenia, no difference were observed between the two groups in SPPB score, crude appendicular lean mass (ALM) and ALM/BMI in the age-adjusted model (Table 1).

### Baseline to follow-up changes

#### Muscle mass, physical performance and clinical data

Tables 2 and 3 shows comparisons of muscle mass, physical performance and clinical end-points at baseline and follow-up. At follow-up, muscle mass was significantly reduced if compared to baseline (ALM and ALM/BMI *p* < 0.001), both in general population (Table 2) and in the two groups (Table 3).

**Table 2:** Muscle mass, physical performance and clinical end-points at baseline and follow-up in all subjects

	Baseline (mean ± sd; median [Q1-Q3])	Follow-up (mean ± sd; median [Q1-Q3])	*p*-value
BMI (kg/m^2^)	27.4 ± 4.8	27.2 ± 4.7	n.s.
*ALM (kg)	16.7 ± 3.7	16.1 ± 3.3	**< 0.001**
*ALM/BMI	0.61 ± 0.13	0.60 ± 0.11	**< 0.001**
SPPB (total score)	7 [7–7.5]	9 [7–10]	**< 0.001**
Medical events (n°)	49 [47–51]	50 [47–52]	**< 0.001**
Therapy (%)			
*Beta-blockers*	40.2	31.7	n.s.
*ACE inhibitors*	30.5	19.5	**0.012**
*ARB*	23.2	12.2	**0.012**
*Diuretics*	24.4	22.0	n.s.
*Other medications* ^*^	22.0	24.4	n.s.

*Other medications are related to cardiovascular system. *p* values refer to comparisons between baseline and follow-up values. “n.s.” indicates non-significant differences (*p* > 0.05). Statistically significant differences (*p* < 0.05) are highlighted. Paired statistical tests were used as appropriate for continuous and categorical variables

**Table 3:** Comparison of muscle mass and BMI between baseline and follow-up in the two groups

	MCI (mean ± sd; median [Q1-Q3])			HALE (mean ± sd; median [Q1-Q3])		
	Baseline	Follow-up	*p*-value	Baseline	Follow-up	*p*-value
BMI (kg/m^2^)	28.8 ± 5.2	28.3 ± 5.2	0.169	25.6 ± 5.2	25.7 ± 3.6	0.715
ALM (kg)	17.0 ± 4.2	16.0 ± 3.3	**< 0.001**	16.4 ± 3.0	16.2 ± 3.4	**< 0.001**
ALM/BMI	0.59 ± 0.1	0.57 ± 0.1	**0.042**	0.64 ± 0.1	0.63 ± 0.1	**< 0.001**
SPPB (total score)	7 [7–7.3]	10 [7–10]	**< 0.001**	7 [7–8]	8 [7–10.5]	**0.001**

*ALM *Appendicular lean mass; *BMI* Body Mass Index; *SPPB* Short Physical Performance Battery. *p* values refer to within-group comparisons between baseline and follow-up. Statistically significant differences (*p* < 0.05) are highlighted. Paired statistical tests were used as appropriate

No significant changes in BMI were observed across the study period, even if MCI participants showed higher BMI compared to the HALE ones, both at baseline and follow-up (Tables 2 and 3). Physical performance improved, as the median SPPB score changed from 7 [7–7.5] to 9 [7–10] at follow-up (*p* < 0.001) in the general population (Table 2), with a greater increase in MCI (Table 3). The number of medical events significantly increased after 24 months (*p* < 0.001), and a lower prescription of ACE inhibitors and ARB (*p* = 0.012 for both medications) was observed (Table 2).

#### The cardio-sarcopenia syndrome: intervention effect and relationship between muscle and cardiac mass

**Fig. 1:**
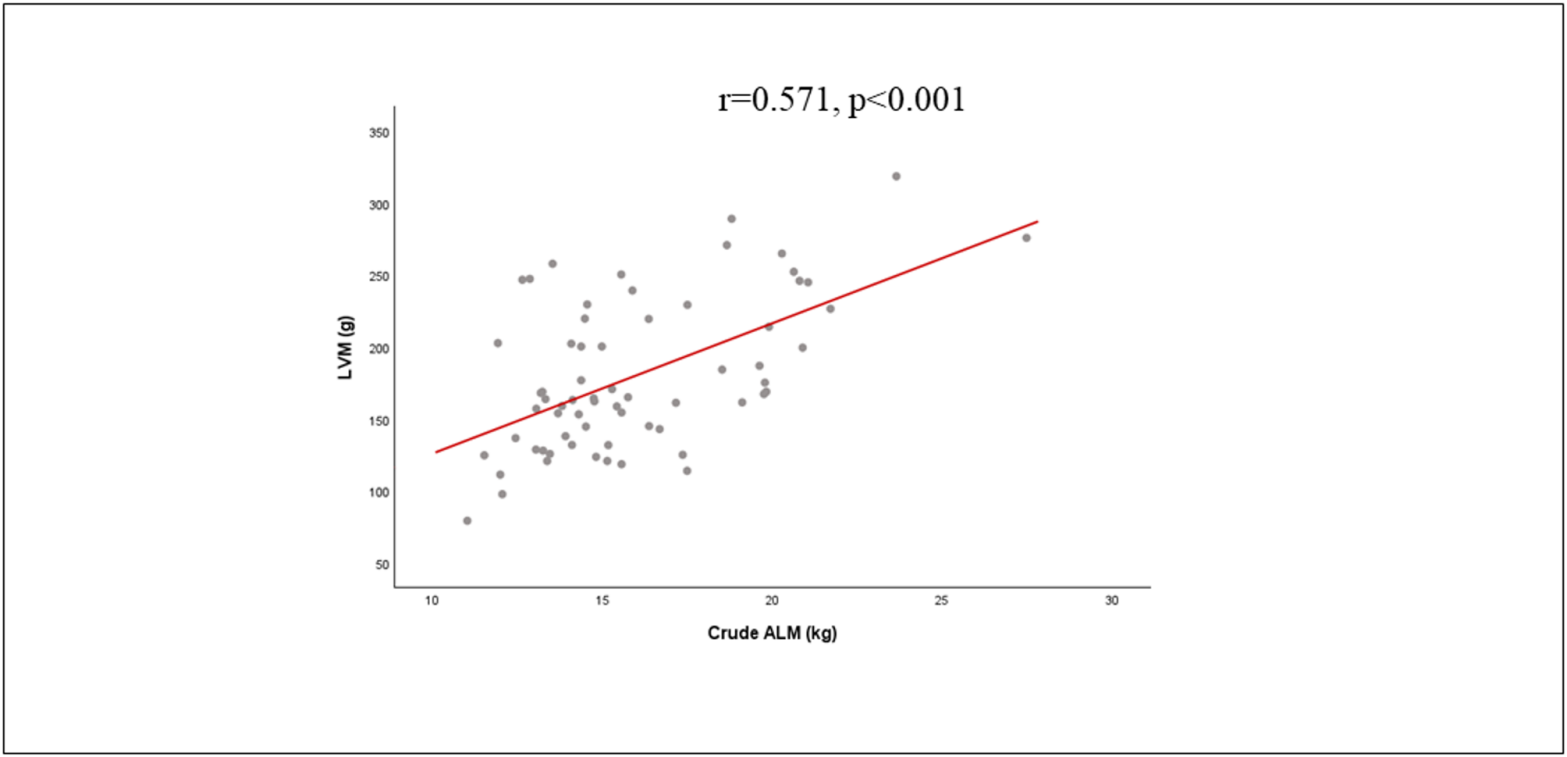
Correlation between left ventricular mass (LVM) and appendicular lean mass (ALM)

Table 4 shows time, type of intervention and interaction factor effects on ALM and ALM/BMI. Two years of follow-up significantly affected ALM, which was reduced in both groups. Time, group and interaction time*group showed significant effects on ALM/BMI with a lower reduction in MCI. We did not observe significant effects of time, type of intervention and interaction factor on cardiac mass, by means of LVM and LVM/body surface area (BSA) (Table 4). At follow-up, a significant correlation between ALM and LVM (*r* = 0.571, *p* < 0.001) was confirmed, consistent with baseline findings and supporting the hypothesis of a cardiac -skeletal muscle axis (Fig. 1) [2].

**Table 4:** Time, intervention and interaction factor effect on skeletal and cardiac mass (unadjusted model; MCI group *n* = 45, HALE group *n* = 35)

	Baseline		Follow-up				
	MCI mean ± sd	HALE mean ± sd	MCI mean ± sd	HALE mean ± sd	Time* *Group*	*Time*	*Group*
ALM (kg)	16.58 ± 0.60	16.79 ± 0.70	16.00 ± 0.54	16.18 ± 0.63	0.930	**< 0.001**	0.819
ALM/BMI	0.58 ± 0.02	0.66 ± 0.02	0.57 ± 0.02	0.63 ± 0.02	**0.019**	**< 0.001**	**0.012**
LVM (gr)	178.8 ± 7.74	186.6 ± 8.77	177.5 ± 7.59	187.0 ± 8.60	0.797	0.901	0.435
LVM/BSA	103.2 ± 3.91	111.1 ± 4.41	103.0 ± 3.77	112.0 ± 4.26	0.776	0.867	0.127

*ALM* Appendicular lean mass; *BMI* Body Mass Index; *BSA* Body surface area; *LVM* Left Ventricular Mass; *SPPB* Short Physical Performance Battery; *SD* standard deviation. *p* values are derived from mixed-effects ANOVA models. The columns indicate: Time × Group - interaction effect, i.e., whether the change over time differs between MCI and HALE groups; Time - main effect of time across all participants; Group - main effect of group regardless of time. Statistical significance is considered at *p* < 0.05

However, after adjusting the analysis for age, BMI, and SBP, these effects were not significant anymore (data not shown).

#### Left and right ventricular remodeling

Tables 5 and 6 shows the time, intervention and interaction factor crude effects on left and right (structural and functional) ventricular echocardiographic parameters.

**Table 5:** Left ventricular echocardiographic data and comparison between the two groups at baseline and follow-up (unadjusted model; MCI *n* = 46, HALE = 36)

	Baseline		Follow-up		Mixed effects ANOVA (*p*)		
Variable	**MCI**	**HALE**	**MCI**	**HALE**	Interaction (time*group)	Time	Group
EDV (ml)	90 *±* 20	88 *±* 23	80 *±* 20	81 *±* 18	0.524	**< 0.001**	0.904
ESV (ml)	27 *±* 8	27 *±* 9	25 *±* 7	24 *±* 8	0.617	**0.003**	0.968
RWT	0.41 *±* 0.07	0.41 *±* 0.07	0.39 *±* 0.06	0.39 *±* 0.06	0.759	**0.034**	0.812
FS (%)	41 *± +* 6	40 *±* 5	39 *±* 5	42 *±* 5	**0.004**	0.889	0.402
EF (%)	70 *±* 5	69 *±* 4	70 *±* 5	71 *±* 5	0.117	0.488	0.829
CO (ml)	64 *±* 16	60 *±* 16	57 *±* 15	56 *±* 13	0.313	**< 0.001**	0.457
E _pv_ (cm/sec)	58 + 16	53 *±* 14	54 *±* 13	56 *±* 15	0.082	0.887	0.576
A _pv_ (cm/sec)	82 *±* 19	79 *±* 16	78 *±* 19	76 *±* 15	0.619	**0.026**	0.477
E/A _pv_	0.68 *±* 0.15	0.69 *±* 0.18	0.68 *±* 0.15	0.75 *±* 0.22	0.082	0.124	0.289
DTE S_pv_ (cm/sec)	7.30 *±* 1.43	7.17 *±* 1.34	6.4 *±* 0.99	6.6 *±* 1.04	0.465	**< 0.001**	0.958
DTE S_tvi_ (cm)	1.54 *±* 0.32	1.49 *±* 0.21	1.36 *±* 0.20	1.38 *±* 0.21	0.311	**< 0.001**	0.826
DTE E’_pv_ (cm/sec)	6.51 *±* 2.02	5.64 *±* 1.34	5.45 *±* 1.20	5.77 *±* 1.15	**0.011**	**0.042**	0.335
DTE E’_tvi_ (cm)	0.77 *±* 0.26	0.68 *±* 0.18	0.65 *±* 0.13	0.67 *±* 0.13	**0.043**	0.127	0.280
E/E’	8.83 *±* 2.31	9.09 *±* 2.25	9.94 *±* 2.92	9.61 *±* 2,67	0.204	0.139	0.590

*p* values are derived from mixed-effects ANOVA models. The columns indicate: Interaction (time × group) - whether the change over time differs between groups; Time - whether there is a significant change over time across all subjects; Group - whether there is a significant difference between MCI and HALE groups regardless of time. Statistical significance is considered at *p* < 0.05

**Table 6:** Right ventricular functional echocardiographic data and comparison between the two groups at baseline and follow up (unadjusted model; MCI *n* = 46, HALE = 36)

	Baseline		Follow-up		Mixed effects ANOVA (*p*)		
Variable	**MCI**	**HALE**	**MCI**	**HALE**	Interaction (time*group)	Time	Group
E_pv_ (cm/sec)	42 *±* 11	38 *±* 7	40 *±* 11	35 *±* 8	0.583	**0.048**	**0.024**
A_pv_ (cm/sec)	41 *±* 9	42 *±* 11	38 *±* 9	38 *±* 8	0.322	**0.05**	0.774
E/A _pv_	1.04 + 0.27	0.97 *±* 0.33	1.03 *±* 0.23	0.98 *±* 0.22	0.97	0.76	0.16
DTE S_pv_ (cm/sec)	14.5 *±* 4.2	15.0 *±* 3.4	13.4 *±* 3.4	12.4 *±* 2.9	0.194	**0.02**	0.695
DTE S_tvi_ (cm)	2.72 *±* 0.80	2.90 *±* 0.66	2.50 *±* 0.61	2.59 *±* 0.59	0.796	**0.035**	0.266
DTE E’_pv_ (cm/sec)	11.3 *±* 3.2	10.6 *±* 2.4	9.7 *±* 2.7	9.3 *±* 2.0	0.770	**0.002**	0.186
DTE E’_tvi_ (cm)	1.70 *±* 0.71	1.88 *±* 0.40	1.57 *±* 0.36	1.65 *±* 0.40	0.545	**0.029**	0.139
E/E’	3.7 *±* 1.0	3.8 *±* 1.2	4.6 *±* 1.8	3.8 *±* 1.2	0.070	**0.055**	**0.034**

Data are presented as mean *±* standard deviation. *p* values are derived from mixed-effects ANOVA models. The columns indicate: Interaction (time × group) - whether the change over time differs between groups; Time - whether there is a significant change over time across all subjects; Group - whether there is a significant difference between MCI and HALE groups regardless of time. Statistical significance is considered at *p* < 0.05

*A* end-diastolic wave; *CO* cardiac output; *DTE* Doppler tissue echocardiography; *E* proto-diastolic wave; *E’* proto-diastolic myocardial wave; *EDV* end-diastolic volume; *EF* ejection fraction; *ESV* end-systolic volume; *FS* fractional shortening; *pv* peak velocity; *RWT* relative wall thickness; *S* systolic myocardial wave; *tvi* time-velocity integral

Regarding left ventricle (LV), type of intervention, exercise or lifestyle education, did not influence structural and functional parameters. A significant influence of the time factor was observed for volumes, cardiac output (CO), relative wall thickness (RWT), systolic (S wave) and diastolic (E’) myocardial tissue Doppler parameters suggesting a progressive impairment of LV systolic and diastolic function during the follow-up period (Table 5). A mild and non-significant increase of the ratio of early diastolic myocardial inflow velocity to E’ velocity (E/E’) was observed in both groups after two-year intervention indicating the trend to the progression in LV filling pressure (Table 5). Conventional systolic parameters, such as ejection fraction (EF) and fractional shortening (FS) did not show effects of time and type of intervention (Table 5).

The effects of aging were also detected on the right ventricular (RV) function: at follow-up, S and E’ waves, obtained analyzing tricuspidal annulus excursion, were reduced compared to baseline, suggesting a progressive impairment of RV systolic and diastolic function regardless the intervention (Table 6).

The ANOVA model, adjusted for age, BMI, SBP and sex (analysis of covariance, ANCOVA), showed that time effect was significant only for S wave of RV (*p* = 0.038) (data not shown). A significant time-group interaction was observed only for LV and FS, which increased in MCI and decreased in HALE (*p* = 0.004), indicating that the left myocardial performance trend was independent by time and appeared improved in exercised group (data not shown).

## Discussion

This study showed that a two-year multicomponent intervention failed to induce significant structural or functional cardiac changes in frail, sarcopenic older adults. We did not observe significant differences due to intervention on LV remodeling and in systolic or diastolic functions of RV and LV.

The sample considered in the present study is composed by subjects enrolled at the Frailty Clinic of University-Hospital of Parma site for the SPRINTT project (CARDIOSPRINTT) [2, 12].

The SPRINTT randomized controlled trial aimed to evaluate the efficacy of a multicomponent treatment, based on moderate intensity physical exercise and nutritional counseling (1.1–1.2 g/kg of protein daily intake) on mobility-disability incidence prevention, compared to a lifestyle education program in physically frail, sarcopenic older adults. The heart was not the primary target of the trial, which focused on the risk of motor disability. This goal was successfully achieved, as the control group exhibited a higher rate of mobility disability (52.7%) compared to the intervention group (46.8%, *p* < 0.005) [10].

In our population, MCI showed a greater improvement in SPPB compared with HALE confirming the results of the entire trial.

The CARDIOSPRINTT is an ancillary study focused on evaluation of cardiac adaptations in a population at high cardiovascular risk, in order to identify prevention strategies to reduce cardiovascular mortality. This target is not necessarily far from the main outcome of the original trial, given the existence of a close correlation between CV diseases, sarcopenia and frailty [2]. This is the reason why sarcopenia and frailty have been proposed as new CV risk factors in geriatric patients [13–15].

In a previous published work, we both evaluated at baseline the cardiac adaptations of the frail, sarcopenic older individuals and identified the determinants of left ventricular mass [12].

In addition, we demonstrated in the follow-up data at 24 months a significant correlation between LVM and SMM confirming the existence of the cardio-sarcopenia syndrome [2].

In the present paper, we longitudinally analyzed the impact of the two-year multimodal intervention on the cardio-sarcopenia syndrome, and did not find significant effects on the heart in the MCI compared with HALE.

To our knowledge, the effect of physical activity on cardiovascular structure and function in older people with frailty and sarcopenia has never been thoroughly investigated. The present study aims to fill the gaps on this topic and this represents a strength. In addition, the sample size, albeit not very numerous, was homogeneous for age, with physical frailty defined as a SPPB score between 3 and 9 and low ALM, as required by SPRINTT protocol [8, 9].

Several hypotheses may explain why the intervention failed to affect cardiac outcomes: first of all, the intensity of the exercise, designed in the LIFE study and adopted in SPRINTT, to prevent disability, was probably not enough to induce changes in the cardiac parameters within two years [11]. Furthermore, it is possible that the length of the study was not sufficient to determine long-term effect of physical exercise on the CV system. We should also acknowledge the wide variability of multimorbidity in this population, and the already mentioned different goals of SPRINTT and CARDIOSPRINTT [8–10]. Compared to the main SPRINTT trial, our sub-study aimed to test the effects of the exercise protocol on cardiac structure and function and not just the prevention of disability. These findings suggest that exercise programs should be tailored to the individual’s degree of functional impairment—more intensive for mildly frail, and lighter for severely physically impaired subjects.

However, the longitudinal analysis of this study clearly demonstrated the effects of the CV aging process in both ventricles [16, 17]. We observed significant and group-independent variations over time, of LV structure such as a significant decrease in end- diastolic (EDV) and end-systolic (ESV) volumes (respectively p < 0.001 and p = 0.003). We hypothesized that all these changes were the consequences of the progression of diastolic dysfunction, concept well-supported by the reduction of LV E’ wave (E_pv_: *p* = 0.042).

The impaired LV relaxation resulted in lower ventricular filling, i.e. preload, and thus LV volumes, and CO reduction (*p* < 0.001). However, LVM did not significantly change at follow-up compared with baseline.

Regarding LV systolic function, EF, a well-known pump function parameter, did not change but myocardial systolic waves (S_pv_: *p* < 0.001; S_tvi_: *p* < 0.001) were reduced over time suggesting a progression of systolic dysfunction. All these findings are in accordance with the concept of cardiac aging [16, 17].

The effects of aging were confirmed at the RV analysis by a reduction of S and E’ myocardial waves confirming the progressive impairment of systolic and diastolic function involving also this chamber.

Unexpectedly, RWT, an index of LV geometry, slightly decreased (*p* = 0.034) at follow-up, as would be expected in view of the aging progression of diastolic dysfunction leading to a change from eccentric to concentric remodeling [16]. This finding will require confirmation by future studies.

After adjusting for sex, BMI, SBP and age, almost all differences due to the time disappeared. However, a significant effect due to the time-group interaction was observed on LV for FS which increased in MCI and decreased in HALE (*p* < 0.001) suggesting an improvement of systolic function in the active group.

Although we did not show significant effects on cardiac remodeling and LVM induced by physical exercise, physical performance globally improved, as the median SPPB score changed from 7 [7–7.5] to 9 [7–10] in the general population with a greater increase in MCI group.

The cardiac -skeletal muscle axis was also analyzed longitudinally, and we confirmed the close correlation between LVM and SMM (*r* = 0.571, *p* < 0.001), previously demonstrated in the cross-sectional part of the CARDIOSPRINTT [2].

The cardio-sarcopenia syndrome, as firstly described by Keng et al. [1], is a novel entity in which a reduction in mass coexists in the skeletal and cardiac muscle. Compared with the study of Keng et al. [1], we assessed skeletal mass with DXA instead of BIA.

These results seem to disagree with data reported in the literature that have shown an increase in LV thicknesses and LVM in aging, changes attributed to cardiac adaptations resulting from increased aortic stiffness and consequent increase in systolic pressure. However, these observations were detected in a large population independently from other concomitant syndromes including sarcopenia.

It must be underlined that we demonstrated the existence of cardiac- skeletal muscle axis in the specific setting of sarcopenic and frail older adults not receiving much attention in the current literature. The specific setting and characteristics of the patients do not allow the translation of these results in all older people [1, 2]. However, after further confirmation in populations different from the CARDIOSPRINTT, the cardiac-skeletal muscle axis could become a specific target to multidomain interventions addressing cardiovascular aging. According to these preliminary results, sarcopenia should be added as a marker of heart’s health, highlighting the need for cardiovascular screening in sarcopenic patients.

Despite the potential strengths of the study, we should also acknowledge some limitations. First, the small number of participants evaluated in the CARDIOSPRINTT; second, the not optimal adherence to the study in the MCI: of the 46 subjects, more than 15% did not complete the entire program or attend it continuously. Several factors may have contributed to reduced adherence in the MCI group, including neuropsychological issues, social burdens, physical comorbidities (e.g., obesity, osteoarticular disease), and caregiving responsibilities.

## Conclusion

This study demonstrated that a multicomponent intervention based on physical activity and nutritional counselling, administered for 24 months, was unable to significantly modify the structure and function of the heart, compared to a healthy ageing lifestyle education program.

These results suggest that the treatment based on physical exercise must be personalized according to the degree of mobility impairment to obtain substantial effects on cardiovascular health [18–20].

Cardiovascular preventive interventions should be performed earlier than the onset of physical and muscle impairment to preserve the cardiac-skeletal muscle axis.

## Supplementary Information

Supplementary Material 1.

Supplementary Material 2.

## Data Availability

No datasets were generated or analysed during the current study.

## Abbreviations

A’: Late diastolic myocardial wave
ALM: Appendicular lean mass
ARB: Angiotensin II receptor blockers
BMI: Body mass index
BP: Blood pressure
BSA: Body Surface Area
CAD: Coronary artery disease
CARDIOSPRINTT: Ancillary study of SPRINTT
CO: Cardiac output
COPD: Chronic obstructive pulmonary disease
CV: Cardiovascular
DBP: Diastolic BP
DTE: Doppler Tissue Echocardiography
E’: Early diastolic myocardial wave
EDD: End diastolic diameter
EDV: End diastolic volume
EF: Ejection fraction
ESD: End systolic diameter
ESV: End systolic volume
FNIH: National Institutes of Health
FS: Fractional Shortening
Hale: Healthy aging lifestyle education
HR: Heart rate
LV: left ventricle
LVH: LV hypertrophy
LVM: left ventricular mass
LVM/BSA: LVM indexed to body surface area
MCI: Multi-component intervention
MMSE: Mini Mental State Examination test
pv: Peak flow velocity
PWT: Posterior wall thickness
RV: Right ventricle
RWT: Relative wall thickness
S: Systolic myocardial wave
SBP: Systolic blood pressure
SMM: Skeletal muscle mass
SPPB: Short Physical Performance Battery
SPRINTT: Sarcopenia and Physical Frailty in Older People: Multicomponent Treatment Strategies Trial
SWT: Septal wall thickness
tvi: Time velocity integral
