# Supplementary material for: Effects of two-year adapted physical exercise program and nutritional counselling on cardio-sarcopenia syndrome in older adults with low muscle function
Source: Eur Rev Aging Phys Act. 2025 Jul 4;22:11. doi: 10.1186/s11556-025-00377-8 (PMC12231304; doi:10.1186/s11556-025-00377-8)
Supplement: Supplementary file 2 — Supplementary Material 2. [file 11556_2025_377_MOESM2_ESM.docx]

**Supplementary Information**

**Study intervention**

The clinical trial evaluated the effect of a multi-component intervention (MCI) versus a healthy aging lifestyle education (HALE) program on the risk rate of incident motor disability in frail and sarcopenic elderly people. Interventions were administered for up to 24 months [8-10].

MCI program: The intervention consists of a combination of moderate intensity physical activity and nutritional counseling/dietary intervention, with ICT support.

The physical activity component is based on the exercise protocol implemented in the LIFE study, with moderate intensity aerobic, strength, flexibility and balance training sessions [11].

Aerobic exercise consisted of a 20-minute walk at an intensity (Rate of Perceived Exertion) RPE = 13 (somewhat hard) progressively increased to 40 minutes in the first 6 months. Other forms of endurance activity (e.g., stationary cycling) may be utilized on a limited basis when regular walking is medically or behaviorally contraindicated.

Each session is preceded by a brief warm-up and followed by a brief cool-down period. In light of current clinical guidelines, participants are instructed to complete flexibility exercises following each bout of walking. Moreover, two times per week, following a bout of walking, participants are instructed during the initial phase of the program to complete a 10-min routine focused on strength exercises for lower extremity muscle groups using adjustable ankle weights. This is followed by a brief lower extremity stretching routine. Balance training is introduced during the initial phase of the program as a complement to the aerobic and strength components. Supplementary instructional materials are provided to participants to reinforce the physical activity training occurring during center-based sessions, so that it can be generalized to the home environment. The intervention also involves encouraging participants to increase all forms of physical activity throughout the day (e.g., leisure sports, gardening, use of stairs as opposed to escalators/elevators, and leisurely walks with friends).

Participants are introduced to the physical activity intervention in a structured way, such that they begin at lighter intensity and gradually increase the intensity over the first 2–3 weeks of the intervention.

Lower extremity strength exercises are performed at an intensity of 15 to 16 on the Borg’s scale for the strength training component of the program (“hard”).

The physical activity program is designed to be performed both at the center and at home. During the intervention, participants train at the center twice a week under direct supervision of instructors. The supervised setting allows instructors to better tailor the program to individual needs and abilities, so as to prevent the early dropout and facilitate the building of self-efficacy and support, which are key to maintaining physical activity over the long term.

Center-based sessions are supplemented, in a progressive fashion, with home-based exercises as a means of facilitating physical activity in multiple settings, adopting healthier behavior, and promoting long-term adherence.

HALE program: Participants allocated to the control group are offered a health educational program. The HALE groups (approximately 10–20 participants per group) meet once or twice a month, with required participation at least once per month. The program is based on a workshop series concerning a variety of topics of relevance to older adults (e.g., recommended vaccinations, management of acute and chronic pain, urinary incontinence, constipation, and diarrhea, etc.). As undertaken in the LIFE study^11^, the program also includes a short instructor-led routine (5–10 min) of upper extremity stretching exercises or some relaxation techniques that will be performed at the end of each class. The rationale for this “placebo exercise” activity is that it helps foster adherence to this arm of the study and increases the perceived benefit of the HALE workshop series to the participants without directly affecting the major study outcomes.

**Inclusion Criteria**

Inclusion criteria for the study was: men and women aged 70 years and older, presence of low according with results from a Dual X-ray absorptiometry scan, in agreement with the Foundation for the National Institutes of Health (FNIH) and physical frailty, assessed as Short Physical Performance Battery (SPPB) with a score in the range between 3 and 9 [8-10]. Participants needed to have sufficient cognitive abilities measured using Mini Mental State Examination test (MMSE), and those with MMSE ≥ 24 were included in the study [8-10].

**Echocardiography**

M-mode, two-dimensional, and Doppler Echocardiography were performed by an ultrasonography-experienced cardiologist (GP), using a commercially available, multi-hertz sector, 2-4 MHz probe-equipped machine (Vivid S5, GE Healthcare, USA). The interventricular septal (SWT) and posterior wall (PWT) thicknesses, systolic (ESD) and diastolic (EDD) diameters, systolic (ESV) and diastolic (EDV) left ventricular (LV) volumes, absolute LVM and indexed to body surface area (LVM/BSA) were calculated as previously described. LV hypertrophy (LVH) was defined as LVM/BSA of >95 g/m^2^ in women and >115 g/m^2^ in men. Relative wall thickness (RWT) was calculated as: (SWT+PWT)/EDD, using the 0.42 cut-off to define eccentric (<0.42) or concentric (>0.42) remodeling [12,13]. Simpson’s biplane rule-based end-diastolic (EDV) and systolic (ESV) volumes and ejection fraction (EF) were calculated, while Fractional Shortening (FS) was: [(EDV – ESV)/EDV] x 100. Cardiac output (CO) was derived by the formula: EDV-ESV.

Mitral inflow pattern was analyzed from apical 4-chamber view and E and A wave and their ratio were considered as peak flow velocity (pv) and time velocity integral (tvi), in order to evaluate the conventional diastolic function. From the same projection, DTE analysis was performed at lateral site and postero-septum of mitral annulus to assess myocardial systolic (S) and diastolic (E’, A’) waves of LV. The ratio of early diastolic mitral inflow velocity to early diastolic mitral annulus velocity (E/E' ratio) was calculated for the estimation of LV filling pressure.

The tricuspidal inflow pattern was analyzed to assess right ventricular diastolic function. At the lateral site of tricuspidal annulus from apical 4-chamber view myocardial S, E’ and A’ were calculated to evaluate the longitudinal motion of RV. E/E' was calculated in order to estimate LV and RV filling pressure.

**Gait Speed on 4-meters distance**

Gait speed was estimated by the time measured by a subtask of Short Physical Performance Battery (SPPB). According to Guralnik JM et al *(N Engl J Med*. 1995;**332**(9):556-561*)*, SPPB includes three subtests evaluating balance, usual gait speed over a 4-meter walk, and time to rise 5 times from a chair without help of upper limbs. Participants were allowed to use a cane if necessary, but not the assistance of another person or other devices. The faster of two walks is used to compute walking speed.

**Medical Events**

The number of the medical history events have been recorded at the enrolling phase and it was considered as a proxy of number of comorbidities. To update the clinical condition of subjects at follow-up, adverse events occurred during the study have been additionally computed. Categories of considered medical events are the following: cardiovascular (cardiac disorders, vascular disorders); diabetes (including metabolism and nutrition disorders; neurologic (nervous system disorders); cancer (neoplasms benign, malignant and unspecified, including cysts and polyps); musculoskeletal (musculoskeletal and connective tissue disorders); arthritis; abdominal (gastrointestinal disorders, renal and urinary disorders); lung (respiratory, thoracic and mediastinal disorders); emotional or psychological (psychiatric disorders); falls; others (blood and lymphatic system disorders, ear and labyrinth disorders, infections and infestations, skin and subcutaneous tissue disorders, surgical and medical procedures, injury, poisoning and procedural complications, general disorders and administration site conditions).

**Medications**

Pharmacological therapy considered in the present study included agents (both plain and in combination) such as beta blocking, ace inhibitors, angiotensin II receptor blockers, diuretics (high and low ceiling) and other medications. The latter included the following drug categories: antiadrenergic agents, centrally and peripherally acting, antiarrhythmics (class I and III), cardiac glycosides, dopaminergic agents, other cardiac preparations, potassium-sparing agents, selective calcium channel blockers with direct cardiac effects, selective calcium channel blockers with mainly vascular effect, and vasodilators used in cardiac diseases.
